# On the *α*-*q*-Mutual Information and the *α*-*q*-Capacities

**DOI:** 10.3390/e23060702

**Published:** 2021-06-01

**Authors:** Velimir M. Ilić, Ivan B. Djordjević

**Affiliations:** 1 Mathematical Institute of the Serbian Academy of Sciences and Arts, Kneza Mihaila 36, 11000 Beograd, Serbia; 2 Department of Electrical and Computer Engineering, University of Arizona, 1230 E. Speedway Blvd., Tucson, AZ 85721, USA; ivan@email.arizona.edu

**Keywords:** rényi entropy, tsallis entropy, landsberg—vedral entropy, gaussian entropy, sharma—mittal entropy, *α*-mutual information, *α*-channel capacity

## Abstract

The measures of information transfer which correspond to non-additive entropies have intensively been studied in previous decades. The majority of the work includes the ones belonging to the Sharma–Mittal entropy class, such as the Rényi, the Tsallis, the Landsberg–Vedral and the Gaussian entropies. All of the considerations follow the same approach, mimicking some of the various and mutually equivalent definitions of Shannon information measures, and the information transfer is quantified by an appropriately defined measure of mutual information, while the maximal information transfer is considered as a generalized channel capacity. However, all of the previous approaches fail to satisfy at least one of the ineluctable properties which a measure of (maximal) information transfer should satisfy, leading to counterintuitive conclusions and predicting nonphysical behavior even in the case of very simple communication channels. This paper fills the gap by proposing two parameter measures named the α-*q*-mutual information and the α-*q*-capacity. In addition to standard Shannon approaches, special cases of these measures include the α-mutual information and the α-capacity, which are well established in the information theory literature as measures of additive Rényi information transfer, while the cases of the Tsallis, the Landsberg–Vedral and the Gaussian entropies can also be accessed by special choices of the parameters α and *q*. It is shown that, unlike the previous definition, the α-*q*-mutual information and the α-*q*-capacity satisfy the set of properties, which are stated as axioms, by which they reduce to zero in the case of totally destructive channels and to the (maximal) input Sharma–Mittal entropy in the case of perfect transmission, which is consistent with the maximum likelihood detection error. In addition, they are non-negative and less than or equal to the input and the output Sharma–Mittal entropies, in general. Thus, unlike the previous approaches, the proposed (maximal) information transfer measures do not manifest nonphysical behaviors such as sub-capacitance or super-capacitance, which could qualify them as appropriate measures of the Sharma–Mittal information transfer.

## 1. Introduction

In the past, extensive work has been written on defining the information measures which generalize the Shannon entropy [1], such as the one-parameter Rényi entropy [2], the Tsallis entropy [3], the Landsberg–Vedral entropy [4], the Gaussian entropy [5], and the two-parameter Sharma–Mittal entropy [5,6], which reduces to former ones for special choices of the parameters. The Sharma–Mittal entropy can axiomatically be founded as the unique *q*-additive measure [7,8] which satisfies generalized Shannon–Kihinchin axioms [9,10] and which has widely been explored in different research fields starting from statistics [11] and thermodynamics [12,13] to quantum mechanics [14,15], machine learning [16,17] and cosmology [18,19]. The Sharma–Mittal entropy has also been recognized in the field of information theory, where the measures of conditional Sharma–Mittal entropy [20], Sharma–Mittal divergences [21] and Sharma–Mittal entropy rate [22] have been established and analyzed.

Considerable research has also been done in the field of communication theory in order to analyze information transmission in the presence of noise if, instead of Shannon’s entropy, the information is quantified with (instances of) Sharma–Mittal entropy and, in general, the information transfer is quantified by an appropriately defined measure of mutual information, while the maximal information transfer is considered as a generalized channel capacity. Thus, after Rényi’s proposal for the additive generalization of Shannon entropy [2], several different definitions for Rényi information transfer were proposed by Sibson [23], Arimoto [24], Augustin [25], Csiszar [26], Lapidoth and Pfister [27] and Tomamichel and Hayashi [28]. These measures have been explored thoroughly and their operational characterization in coding theory, hypothesis testing, cryptography and quantum information theory was established, which qualifies them as a reasonable measure of Rényi information transfer [29]. Similar attempts have also been made in the case of non-additive entropies. Thus, starting from the work of Daroczy [30], who introduced a measure for generalized information transfer related to the Tsallis entropy, several attempts followed for the measures which correspond to non-additive particular instances of the Sharma–Mittal entropy, so the definitions for the Rényi information transfer were considered in [24,31], for the Tsallis information transfer in [32] and for the Landsber–Vedral information transfer in [4,33].

In this paper we provide a general treatment of the Sharma–Mittal entropy transfer and a detailed analysis of existing measures, showing that all of the definitions related to non-additive entropies fail to satisfy at least one of the ineluctable properties common to the Shannon case, which we state as axioms, by which the information transfer has to be non-negative, less than the input and output uncertainty, equal to the input uncertainty in the case of perfect transmission and equal to zero, in the case of a totally destructive channel. Thus, breaking some of these axioms implies unexpected and counterintuitive conclusions about the channels, such as achieving super-capacitance or sub-capacitance [4], which could be treated as nonphysical behavior. As an alternative, we propose the α-*q*-mutual information as a measure of Sharma–Mittal information transfer, maximized with the α-*q*-capacity. The α-*q* mutual information generalizes the α-mutual information by Arimoto [24], which is defined as a *q*-difference between the input Sharma–Mittal entropy and the appropriately defined conditional Sharma–Mittal entropy if the output is given, while the α-*q*-capacity represents a generalization of Arimoto’s α-capacity in the case of q=1. In addition, several other instances can be obtained by specifying the values of parameters α and *q*, which includes the information transfer measures for the Tsallis, the Landsber–Vedral and the Shannon entropy, as well as the case of the Gaussian entropy which was not considered before in the context of information transmission.

The paper is organized as follows. The basic properties and special instances of the Sharma–Mittal entropy are listed in Section 2. Section 3 reviews the basics of communication theory, introduces the basic communication channels and establishes the set of axioms which information transfer measures should satisfy. The information transfer measures which are defined by Arimoto are introduced in Section 4, and the alternative definitions for Rényi information transfer measures are discussed in Section 5. Finally, the α-*q*-mutual information and the α-*q*-capacities are proposed and their properties analyzed in Section 6 while the previously proposed measures of Sharma–Mittal entropy transfer are discussed in Section 7.

## 2. Sharma–Mittal Entropy

Let the sets of positive and nonnegative real numbers be denoted with $R^{+}$ and $R_{0}^{+}$, respectively, and let the mapping $\eta_{q}:R\to R$ be defined in

(1) $$\eta_{q}\left(x\right)=\left\{\begin{array}{cc} x,\;\; & \mathrm{for}\;q=1\\ \frac{2^{(1-q)\;x}-1}{(1-q)\ln2},\;\; & \mathrm{for}\;q\neq 1 \end{array}\right.$$

so that its inverse is given in

(2) $$\eta_{q}^{-1}\left(x\right)=\left\{\begin{array}{cc} x,\;\;\;\; & \mathrm{for}\;q=1\\ \frac{1}{1-q}\log\left(\left(1-q\right)x\ln2+1\right),\;\; & \mathrm{for}\;q\neq 1 \end{array}\right..$$

The mapping *q* and its inverse are increasing continuous (hence invertible) functions such that 0)=0. The *q*-logarithm is defined in

(3) $${\mathrm{Log}}_{q}\left(x\right)=\eta_{q}\left(\log x\right)=\left\{\begin{array}{cc} \;\log x,\;\; & \mathrm{for}\;q=1\\ \frac{x^{\left(1-q\right)}-1}{(1-q)\ln2},\;\; & \mathrm{for}\;q\neq 1 \end{array}\right.,$$

and its inverse, the *q*-exponential, is defined in

(4) $${\mathrm{Exp}}_{q}\left(y\right)=\left\{\begin{array}{cc} \;2^{y},\;\;\;\; & \mathrm{for}\;q=1\\ {\left(1+(1-q)y\ln2\right)}^{\frac{1}{1-q}}\; & \mathrm{for}\;q\neq 1 \end{array}\right.,$$

for 1+(1−q)yln2>0. Using *η_q_*, we can define the pseudo-addition operation ${⊕}_{q}$ [7,8]

(5) $$x{⊕}_{q}y=\eta_{q}\left(\eta_{q}^{-1}\left(x\right)+\eta_{q}^{-1}\left(y\right)\right)=x+y+\left(1-q\right)xy;\;x,y\in R,$$

and its inverse operation, the pseudo substraction

(6) $$x{⊖}_{q}y=\eta_{q}\left(\eta_{q}^{-1}\left(x\right)-\eta_{q}^{-1}\left(y\right)\right)=\frac{x-y}{1+(1-q)y\ln2};\;x,y\in R.$$

The ${⊕}_{q}$ can be rewritten in terms of the generalized logarithm by settings x=logu and y=logv so that

(7) $${\mathrm{Log}}_{q}\left(u\cdot v\right)={\mathrm{Log}}_{q}\left(u\right){⊕}_{q}{\mathrm{Log}}_{q}\left(v\right);\;u,v\in R_{+}.$$

Let the set of all *n*-dimensional distributions be denoted with

(8) $$\Delta_{n}\equiv \left\{\left(p_{1},\ldots ,p_{n}\right)|\;p_{i}\geq 0,\sum_{i=1}^{n}p_{i}=1\right\};\;n>1.$$

Let the function $H_{n}:\Delta_{n}\to R_{0}^{+}$ satisfy the following the Shannon–Khinchin axioms, for all n∈N, n>1.

GSK1 $H_{n}$ is continuous in $\Delta_{n}$;
GSK2 $H_{n}$ takes its largest value for the uniform distribution, $U_{n}=\left(1/n,\ldots ,1/n\right)\in \Delta_{n}$, i.e., $H_{n}\left(P\right)\leq H_{n}\left(U_{n}\right)$, for any $P\in \Delta_{n}$;
GSK3 $H_{n}$ is expandable: $H_{n+1}\left(p_{1},p_{2},\ldots ,p_{n},0\right)=H_{n}\left(p_{1},p_{2},\ldots ,p_{n}\right)$ for all $\left(p_{1},\ldots ,p_{n}\right)\in \Delta_{n}$;
GSK4 Let $P=\left(p_{1},\ldots ,p_{n}\right)\in \Delta_{n}$, $PQ=\left(r_{11},r_{12},\ldots ,r_{nm}\right)\in \Delta_{nm}$, n,m∈N, n,m>1 such that $p_{i}=\sum_{j=1}^{m}r_{ij}$, and $Q_{|k}=\left(q_{1|k},\ldots ,q_{m|k}\right)\in \Delta_{m}$, where $q_{i|k}=r_{ik}/p_{k}$ and $\alpha \in R_{0}^{+}$ are some fixed parameters. Then,
  (9) $$H_{nm}\left(PQ\right)=H_{n}\left(P\right){⊕}_{q}H_{m}\left(Q|P\right),\;\mathrm{where}\;H_{m}\left(Q|P\right)=f^{-1}\left(\sum_{k=1}^{n}p_{k}^{\left(\alpha \right)}f\left(H_{m}\left(Q_{|k}\right)\right)\right),$$
  where *f* is an invertible continuous function and $P^{\left(\alpha \right)}=\left(p_{1}^{\left(\alpha \right)},\ldots ,p_{n}^{\left(\alpha \right)}\right)\in \Delta_{n}$ is the α-escort distribution of distribution $P\in \Delta_{n}$ defined in
  (10) $$p_{k}^{\left(\alpha \right)}=\frac{p_{k}^{\alpha }}{\sum_{i=1}^{n}p_{i}^{\alpha }},\;k=1,\ldots ,n,\;\alpha >0.$$
GSK5 $H_{2}\left(\frac{1}{2},\frac{1}{2}\right)={\mathrm{Log}}_{q}\left(1\right)$.

As shown in [9], the unique function $H_{n}$, which satisfies [GSK1]-[GSK5], is Sharma–Mittal entropy [6].

In the following paragraphs we will assume that *X* and *Y* are discrete jointly distributed random variables taking values from sample spaces $\left\{x_{1},\ldots ,x_{n}\right\}$ and $\left\{y_{1},\ldots ,y_{m}\right\}$, and distributed in accordance to $P_{X}\in \Delta_{n}$ and $P_{Y}\in \Delta_{m}$, respectively. In addition, the joint distribution of *X* and *Y* will be denoted in $P_{X,Y}\in \Delta_{nm}$ and the conditional distribution of *X* given *Y* will be denoted in $P_{X|Y}=\frac{P_{X,Y}\left(x,y\right)}{P_{Y}\left(y\right)}\in \Delta_{m}$, provided that $P_{Y}\left(y\right)>0$. We will identify the entropy of a random variable *X* with the entropy of its distribution $P_{X}$ and the Sharma–Mittal entropy will be denoted with $H_{\alpha ,q}\left(X\right)\equiv H_{n}\left(P_{X}\right)$.

Thus, for a random variable which is distributed to *X*, Sharma–Mittal entropy can be expressed in

(11) $$H_{\alpha ,q}\left(X\right)=\frac{1}{1-q}\left({\left(\sum_{x}P_{X}{\left(x\right)}^{\alpha }\right)}^{\frac{1-q}{1-\alpha }}-1\right),$$

and it can equivalently be expressed as the *η_q_* transformation of Rényi entropy as in

(12) $$H_{\alpha ,q}\left(X\right)\equiv \eta_{q}\left(R_{\alpha }\left(X\right)\right).$$

Sharma–Mittal entropy, for $\alpha ,q\in R_{0}^{+}\backslash 1$, being a continuous function of the parameters and the sums goes over the support of $P_{X}$. Thus, in the case of q=1, α≠1, Sharma–Mittal reduces to Rényi entropy of order α [2]

(13) $$R_{\alpha }\left(X\right)\equiv H_{\alpha ,1}\left(X\right)=\;\frac{1}{1-\alpha }\log\left(\sum_{x}P_{X}{\left(x\right)}^{\alpha }\right),$$

which further reduces to Shannon entropy for α=1,q=1,^34^]

(14) $$S\left(X\right)\equiv H_{1,1}\left(X\right)=\sum_{x}P_{X}\left(x\right)\log P_{X}\left(x\right),$$

while in the case of q≠1, α=1 it reduces to Gaussian entropy [5]

(15) $$G_{q}\left(X\right)\equiv H_{1,q}\left(X\right)=\frac{1}{(1-q)\ln2}\left(\overset{n}{\underset{i=1}{\Pi }}P_{X}{\left(x\right)}^{P_{X}\left(x\right)}-1\right).$$

In addition, Tsallis entropy [3] is obtained for α=q≠1,

(16) $$T_{q}\left(X\right)\equiv \frac{1}{(1-q)\ln2}\left(\sum_{x}P_{X}{\left(x\right)}^{q}-1\right),$$

while in the case of for q=2−α it reduces to the Landsberg–Vedral entropy [4]

(17) $$L_{\alpha }\left(X\right)\equiv H_{\alpha ,2-\alpha }\left(X\right)=\frac{1}{(\alpha -1)\ln2}\left(\frac{1}{\sum_{x}P_{X}{\left(x\right)}^{\alpha }}-1\right).$$

## 3. Sharma–Mittal Information Transfer Axioms

One of the main goals of information and communication theories is characterization and analysis of the information transfer between sender *X* and receiver *Y*, which communicate through a channel. The sender and receiver are described by probability distributions $P_{X}$ and $P_{Y}$ while the communication channel with the input *X* and the output *Y* is described by the transition matrix $P_{Y|X}$:

(18) $$P_{Y|X}^{\left(i,j\right)}\equiv P_{Y|X}\left(y_{j}|x_{i}\right).$$

We assume that maximum likelihood detection is performed at the receiver, which is defined by the mapping $d:\left\{y_{1},\ldots ,y_{m}\right\}\to \left\{x_{1},\ldots ,x_{n}\right\}$ as follows:

(19) $$d\left(y_{j}\right)=x_{i}\;\Leftrightarrow \;P_{Y|X}\left(y_{j}|x_{i}\right)>P_{Y|X}\left(y_{j}|x_{k}\right);\;\mathrm{for}\;\mathrm{all}\;k\neq i,$$

assuming that the inequality in (19) is uniquely satisfied. Thus, if the input symbol $x_{i}$ is sent and the output symbol $y_{j}$ is received, the $x_{i}$ will be detected if $x_{i}=d\left(y_{j}\right)$ and a detection error will be made otherwise, and we define the error function functions $\phi :\left\{x_{1},\ldots ,x_{m}\right\}\times \left\{y_{1},\ldots ,y_{m}\right\}\to \left\{0,1\right\}$ as in

(20) $$\phi \left(x_{i},y_{j}\right)=\left\{\begin{array}{cc} 1, & \mathrm{if}\;x_{i}=d\left(y_{j}\right)\\ 0, & \mathrm{otherwise}, \end{array}\right.$$

the detection error if a symbol $x_{i}$ is sent

(21) $$P_{err}\left(x_{i}\right)=\sum_{y_{j}}P_{Y|X}\left(y_{j}|x_{i}\right)\phi \left(x_{i},y_{j}\right);\;\mathrm{for}\;\mathrm{all}\;x_{i},$$

as well as the average detection error

(22) $${\bar{P}}_{err}=\sum_{x_{i}}P_{X}\left(x_{i}\right)P_{err}\left(x_{i}\right)=\sum_{x_{i},y_{j}}P_{X,Y}\left(x,y\right)\phi \left(x_{i},y_{j}\right).$$

**Totally destructive channel**: A channel is said to be totally destructive if

(23) $$P_{Y|X}^{\left(i,j\right)}=P_{Y|X}\left(y_{j}|x_{i}\right)=P_{Y}\left(y_{j}\right)=\frac{1}{m};\;\mathrm{for}\;\mathrm{all}\;x_{i},$$

i.e., if the sender *X* and receiver *Y* are described by independent random variables,

(24) $$X⊥\;\;\;⊥Y\;\Leftrightarrow \;P_{X,Y}\left(x,y\right)=P_{X}\left(x\right)P_{Y}\left(y\right),$$

where the relationship of independence is denoted in ⊥⊥. In this case, $\phi_{i}\left(y_{j}\right)=1$ for all $y_{j}$ and the probability of error is $P_{err}\left(x_{i}\right)=1$; for all $x_{i}$, as well as the average probability of error ${\bar{P}}_{err}=1$, which means that a correct maximum likelihood detection is not possible.

**Perfect communication channel**: A channel is said to be perfect if for every $x_{i}$,

(25) $$P_{Y|X}\left(y_{j}|x_{i}\right)>0,\;\mathrm{for}\;\mathrm{at}\;\mathrm{least}\;\mathrm{one}\;y_{j}$$

and for every $y_{j}$

(26) $$P_{Y|X}\left(y_{j}|x_{i}\right)>0,\;\mathrm{for}\;\mathrm{exactly}\;\mathrm{one}\;x_{i}.$$

Note that in this case $P_{Y|X}\left(y_{j}|x_{i}\right)$ can still take a zero value for some $y_{j}$ and that $\phi_{i}\left(y_{j}\right)=0$ for any non-zero $P_{Y|X}\left(y_{j}|x_{i}\right)$. Thus, the error probability is equal to zero $P_{err}\left(x_{i}\right)=0$; for all $x_{i}$, as well as the average probability of error ${\bar{P}}_{err}=0$, which means that perfect detection is possible by means of a maximum likelihood detector.

**Figure 1:**
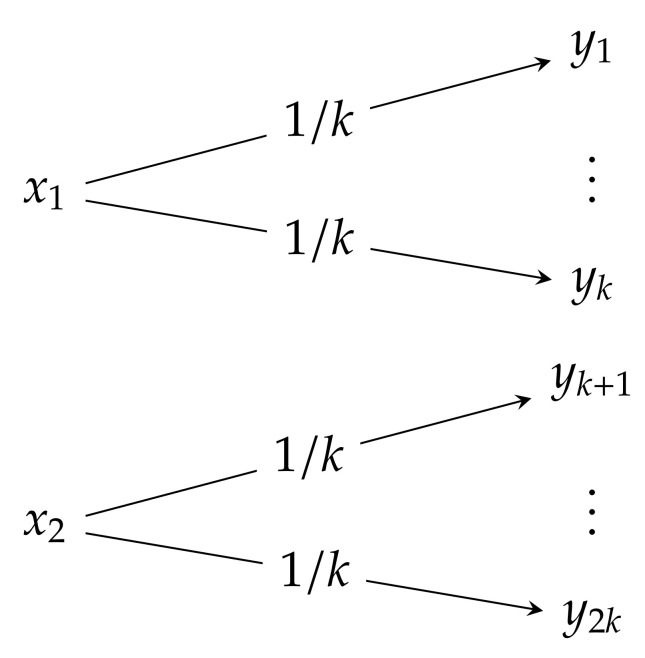
Noisy channel with non-overlapping outputs.

**Noisy channel with non-overlapping outputs**: A simple example of a perfect transmission channel is the noisy channel with non-overlapping outputs (NOC), which is schematically described in Figure 1. It is a 2-input m=2k-output channel (k∈N) defined by the transition matrix:

(27) $$P_{Y|X}=\left[\begin{array}{c} P_{Y|X}\left(\cdot |x_{1}\right)\\ P_{Y|X}\left(\cdot |x_{2}\right) \end{array}\right]=\left[\begin{array}{cccccc} \frac{1}{k} & \ldots & \frac{1}{k} & 0 & \ldots & 0\\ 0 & \ldots & 0 & \frac{1}{k} & \ldots & \frac{1}{k} \end{array}\right]$$

(in this and in the following matrices, the symbol “⋯” stands for the *k*-time repletion). In the case of k=1 and m=2k=2, the channel reduces to the noiseless channel. Although the channel is noisy, the input can always be recovered from the output (if $y_{j}$ is received and j≤k, the input symbol $x_{1}$ is sent, otherwise $x_{2}$ is sent). Thus, it is expected that the information which is passed through the channel is equal to the information that can be generated by the input. Note that for a channel input distributed in accordance with

(28) $$P_{X}=\left[\begin{array}{c} P_{X}\left(x_{1}\right)\\ P_{X}\left(x_{2}\right) \end{array}\right]=\left[\begin{array}{c} a\\ 1-a \end{array}\right];\;0\leq a\leq 1,$$

the joint probability distribution $P_{X,Y}$ can be expressed as in:

(29) $$P_{X,Y}=\left[\begin{array}{cccccc} \frac{a}{k} & \ldots & \frac{a}{k} & 0 & \ldots & 0\\ 0 & \ldots & 0 & \frac{1-a}{k} & \ldots & \frac{1-a}{k} \end{array}\right]$$

and the output distribution $P_{Y}$, which can be obtained by the summations over columns, is

(30) $$P_{Y}={\left[P_{Y}\left(y_{1}\right),\ldots ,P_{Y}\left(y_{m}\right)\right]}^{T}={\left[\frac{a}{k},\ldots ,\frac{a}{k},\frac{1-a}{k},\ldots ,\frac{1-a}{k}\right]}^{T}.$$

**Figure 2:**
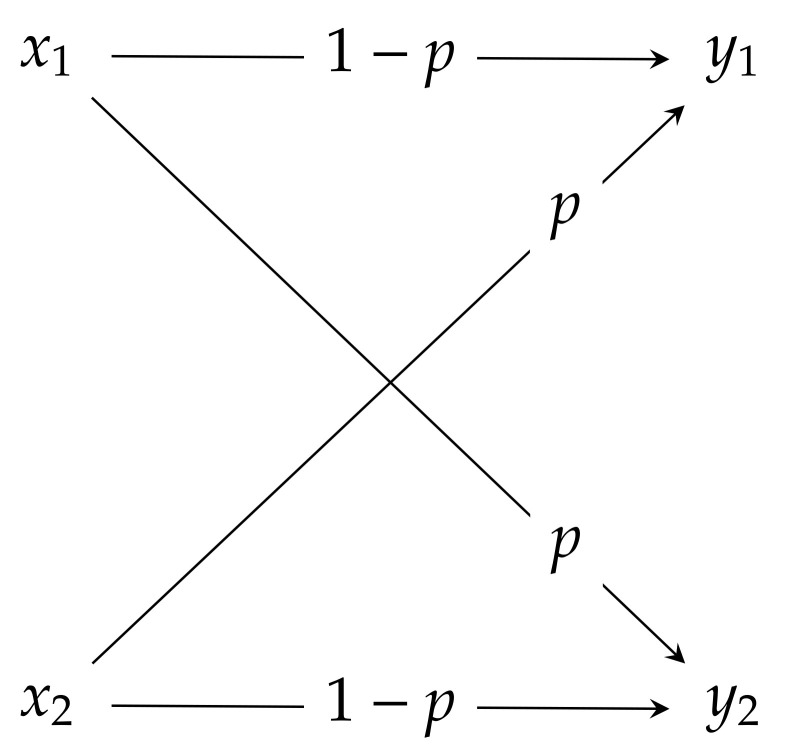
Binary symmetric channel.

**Binary symmetric channels**: The binary symmetric channel (BSC) is a two input two output channel described by the transition matrix

(31) $$P_{Y|X}=\left[\begin{array}{c} P_{Y|X}{\left(\cdot |x_{1}\right)}^{T}\\ P_{Y|X}{\left(\cdot |x_{2}\right)}^{T} \end{array}\right]=\left[\begin{array}{cc} 1-p & p\\ p & 1-p \end{array}\right],$$

which is schematically described in Figure 2. Note that for $p=\frac{1}{2}$ BSC reduces to a totally destructive channel, while in the case of p=0 it reduces to a perfect channel.

### Sharma–Mittal Information Transfer Axioms

In this paper, we search for information theoretical measures of information transfer between sender *X* and receiver *Y*, which communicate through a channel if the information is measured with Sharma–Mittal entropy. Thus, we are interested in the information transfer measure, $I_{\alpha ,q}\left(X,Y\right)$, which is called the α-*q*-mutual information and its maximum,

(32) $$C=\max_{P_{X}}I_{\alpha ,q}\left(X,Y\right),$$

which is called the α-*q*-capacity and which requires the following set of axioms to be satisfied.

(*A*_1_) The channel cannot convey negative information, i.e.,
  (33) $$C_{\alpha ,q}\left(P_{Y|X}\right)\geq I_{\alpha ,q}\left(X,Y\right)\geq 0.$$
(*A*_2_) The information transfer is zero in the case of a totally destructive channel, i.e.,
  (34) $$P_{Y|X}\left(y|x\right)=\frac{1}{m},\;\mathrm{for}\;\mathrm{all}\;x,y\;\Rightarrow \;I_{\alpha ,q}\left(X,Y\right)=C_{\alpha ,q}\left(P_{Y|X}\right)=0,$$
  which is consistent with the conclusion that the average probability of error is one, ${\bar{P}}_{err}=1$, in the case of a totally destructive channel.
(*A*_3_) In the case of perfect transmission, the information transfer is equal to the input information, i.e.,
  (35) $$X=Y\;\Rightarrow \;I_{\alpha ,q}\left(X,Y\right)=H_{\alpha ,q}\left(X\right),\;C_{\alpha ,q}\left(P_{Y|X}\right)={\mathrm{Log}}_{q}n,$$
  which is consistent with the conclusion that the average probability of error is zero, ${\bar{P}}_{err}=0$, in the case of a perfect transmission channel, so that all the information from the input is conveyed.
(*A*_4_) The channel cannot transfer more information than it is possible to be sent, i.e.,
  (36) $$I_{\alpha ,q}\left(X,Y\right)\leq C_{\alpha ,q}\left(P_{Y|X}\right)\leq {\mathrm{Log}}_{q}\;n,$$
  which means that a channel cannot add additional information.
(*A*_5_) The channel cannot transfer more information than it is possible to be received, i.e.,
  (37) $$I_{\alpha ,q}\left(X,Y\right)\leq C_{\alpha ,q}\left(P_{Y|X}\right)\leq {\mathrm{Log}}_{q}\;m,$$
  which means that a channel cannot add additional information.
(*A*_6_) Consistency with the Shannon case:
  (38) $$\lim_{q\to 1,\alpha \to 1}I_{\alpha ,q}\left(X,Y\right)=I\left(X,Y\right),\;\mathrm{and}\;\lim_{q\to 1,\alpha \to 1}C_{\alpha ,q}\left(P_{Y|X}\right)=C\left(P_{Y|X}\right)$$

Thus, the axioms (${\bar{A}}_{2}$) and (${\bar{A}}_{3}$) ensure that the information measures are consistent with the maximum likelihood detection (19)–(21). On the other hand, the axioms (${\bar{A}}_{1}$), (${\bar{A}}_{4}$) and (${\bar{A}}_{5}$), prevent a situation in which a physical system conveys information in spite of going through a completely destructive channel, or in which the negative information transfer is observed, indicating that the channel adds or removes information by itself, which could be treated as nonphysical behavior without an intuitive explanation. Finally, the property (${\bar{A}}_{6}$) ensure that the information transfer measures can be considered as generalizations of corresponding Shannon measures. For these reasons, we assume that the satisfaction of the properties (${\bar{A}}_{1}$)–(${\bar{A}}_{5}$) is mandatory for any reasonable definition of Sharma–Mittal information transfer measures.

## 4. The *α*-Mutual Information and the *α*-Capacity

One of the first proposals for the Rényi mutual information goes back to Arimoto [24], who considered the following definition of mutual information:

(39) $$I_{\alpha }\left(X,Y\right)=\frac{\alpha }{1-\alpha }\log\left(\sum_{y}{\left(\sum_{x}P_{X}^{\left(\alpha \right)}\left(x\right)P_{Y|X}^{\alpha }\left(y|x\right)\right)}^{\frac{1}{\alpha }}\right),$$

where the escort distribution $P_{X^{\left(\alpha \right)}}$ is defined as in (10), and he also invented an iterative algorithm for the computation of the α-capacity [35], which is defined from the α-mutual information:

(40) $$C_{\alpha }\left(P_{Y|X}\right)=\max_{P_{X}}I_{\alpha }\left(X,Y\right).$$

Notably, Arimoto’s mutual information can equivalently be represented using the conditional Rényi entropy

(41) $$R_{\alpha }\left(X|Y\right)=\frac{\alpha }{\alpha -1}\log_{2}\sum_{y}P_{Y}\left(y\right){\left(\sum_{x}P_{X|Y=y}{\left(x\right)}^{\alpha }\right)}^{\frac{1}{\alpha }},$$

as in

(42) $$I_{\alpha }\left(X,Y\right)\equiv R_{\alpha }\left(X\right)-R_{\alpha }\left(X|Y\right),$$

which can be interpreted as the input uncertainty reduction after the output symbols are received and, in the case of α→1, the previous definition reduces to the Shannon case. In addition, this measure is directly related to the famous Gallager exponent

(43) $$E_{0}\left(\rho ,P_{X}\right)=-\log\left(\sum_{y}{\left(\sum_{x}P_{X}\left(x\right)P_{Y|X}^{\frac{1}{1+\rho }}\left(y|x\right)\right)}^{1+\rho }\right),$$

which has been widely used to establish the upper bound of error probability in channel coded communication systems [36] via the relationship [29]

(44) $$I_{\alpha }\left(X,Y\right)=\frac{\alpha }{1-\alpha }E_{0}\left(\frac{1}{\alpha }-1,P_{X}^{\left(\alpha \right)}\right).$$

In addition, in the case of α→1, it reduces to

(45) $$I_{1}\left(X,Y\right)=\lim_{\alpha \to 1}I_{\alpha }\left(X,Y\right)=I\left(X,Y\right),$$

where

(46) $$I\left(X,Y\right)=\sum_{x,y}P_{X,Y}\left(x,y\right)\log\frac{P_{X,Y}\left(x,y\right)}{P_{X}\left(x\right)P_{Y}\left(y\right)}$$

stands for Shannon’s mutual information [37].

The α-mutual information $I_{\alpha }\left(X,Y\right)$ and the α-capacity $C_{\alpha }\left(P_{Y_{X}}\right)$ satisfy the axioms (${\bar{A}}_{1}$)–(${\bar{A}}_{6}$) for q=1 and α>0, as stated by the following theorem, which further justifies their usage as the measures of (maximal) information transfer.

**Theorem** **1.** *The mutual information measures $I_{\alpha }$ and $C_{\alpha }$ satisfy the following set of properties:*

*(A*_1_*)* *The channel cannot convey negative information, i.e.,*
  (47) $$C_{\alpha }\left(P_{Y|X}\right)\geq I_{\alpha }\left(X,Y\right)\geq 0.$$
*(A*_2_*)* *The (maximal) information transfer is zero in the case of a totally destructive channel, i.e.,*
  (48) $$P_{Y|X}\left(y|x\right)=\frac{1}{m},\;\mathrm{for}\;\mathrm{all}\;x,y\;\Rightarrow \;I_{\alpha }\left(X,Y\right)=C_{\alpha }\left(P_{Y|X}\right)=0.$$
*(A*_3_*)* *In the case of perfect transmission, the (maximal) information transfer is equal to the (maximal) input information, i.e.,*
  (49) $$X=Y\;\Rightarrow \;I_{\alpha }\left(X,Y\right)=R_{\alpha }\left(X\right),\;C_{\alpha }\left(P_{Y|X}\right)=\log n.$$
*(A*_4_*)* *The channel cannot transfer more information than it is possible to be sent, i.e.,*
  (50) $$I_{\alpha }\left(X,Y\right)\leq C_{\alpha }\left(P_{Y|X}\right)\leq \log\;n;$$
*(A*_5_*)* *The channel cannot transfer more information than it is possible to be received, i.e.,*
  (51) $$I_{\alpha }\left(X,Y\right)\leq C_{\alpha }\left(P_{Y|X}\right)\leq \log\;m.$$
*(A*_6_*)* *Consistency with the Shannon case:*
  (52) $$\lim_{\alpha \to 1}I_{\alpha }\left(X,Y\right)=I\left(X,Y\right),\;\mathrm{and}\;\lim_{\alpha \to 1}C_{\alpha }\left(P_{Y|X}\right)=C\left(P_{Y|X}\right)$$

**Proof.**  As shown in [38], $R_{\alpha }\left(X|Y\right)\leq R_{\alpha }\left(X\right)$, and the nonnegativity property ($A_{1}$) follows from the definition of Arimoto’s mutual information (42). In addition, if X⊥⊥Y, then $P_{Y|X}\left(y|x\right)=P_{Y}\left(y\right)$ so that the definition (61) implies the property ($A_{2}$). Furthermore, in the case of a perfect transmission channel, the mutual information (61) can be represented in -4.6cm0cm

(53) $$I_{\alpha }\left(X,Y\right)=\frac{\alpha }{\alpha -1}\log\frac{\sum_{y}{\left(\sum_{x}P_{X}{\left(x\right)}^{\alpha }P_{Y|X}^{\alpha }\left(y|x\right)\right)}^{\frac{1}{\alpha }}}{{\left(\sum_{x}P_{X}^{\left(\alpha \right)}\left(x\right)\right)}^{\frac{1}{\alpha }}}=\frac{\alpha }{\alpha -1}\log\frac{\sum_{y}{\left(P_{X}{\left(d\left(y\right)\right)}^{\alpha }P_{Y|X}^{\alpha }\left(y|d\left(y\right)\right)\right)}^{\frac{1}{\alpha }}}{{\left(\sum_{x}P_{X}^{\left(\alpha \right)}\left(x\right)\right)}^{\frac{1}{\alpha }}},$$

and since

(54) $$\begin{array}{c} \;\;\;\sum_{y}{\left(P_{X}{\left(d\left(y\right)\right)}^{\alpha }P_{Y|X}^{\alpha }\left(y|d\left(y\right)\right)\right)}^{\frac{1}{\alpha }}=\sum_{y}P_{X}\left(d\left(y\right)\right)P_{Y|X}\left(y|d\left(y\right)\right)=\\ \sum_{x}\sum_{y:d(y)=x}P_{X}\left(d\left(y\right)\right)P_{Y|X}\left(y|d\left(y\right)\right)=\sum_{x}P_{X}\left(x\right)\sum_{y:d(y)=x}P_{Y|X}\left(y|x\right)=1, \end{array}$$

we obtain $I_{\alpha }\left(X,Y\right)=R_{\alpha }\left(X\right)$, which proves the property ($A_{3}$). Moreover, from the definition as shown in [38], Arimoto’s conditional entropy is positive and satisfies the weak chain rule $R_{\alpha }\left(X|Y\right)\geq R_{\alpha }\left(X\right)-\log m$, so that the properties ($A_{4}$) and ($A_{5}$) follow from the definition of Arimoto’s mutual information (42). Finally, the property ($A_{6}$) follows directly from the equation (45) and can be approved using L’Hôpital’s rule, which completes the proof of the theorem. □

## 5. Alternative Definitions of the *α*-Mutual Information and the *α*-Channel Capacity

Since Rényi’s proposal, there have been several lines of research to find an appropriate definition and characterization of information transfer measures related to Rényi entropy, which are established by the substitution of the Rényi divergence measure

(55) $$D_{\alpha }\left(P||Q\right)=\frac{1}{\alpha -1}\log\left(\sum_{x}P{\left(x\right)}^{\alpha }Q{\left(x\right)}^{1-\alpha }\right),$$

instead of the Kullback–Leibler one,

(56) $$D\left(P||Q\right)=D_{1}\left(P||Q\right)=\sum_{x}P\left(x\right)\log\frac{P(x)}{Q(x)},$$

in some of the various definitions which are equivalent in the case of Shannon information measures (46) [29]:

(57) $$\begin{array}{cc} I(X,Y) & =\min_{Q_{Y}}E\left[D_{\alpha }\left(P_{Y|X}∥Q_{Y}\right)\right]=\min_{Q_{Y}}E\left[D_{\alpha }\left(P_{Y|X}∥Q_{Y}\right)\right]\\ \; & =\min_{Q_{X}}\min_{Q_{Y}}D_{\alpha }\left(P_{X,Y}∥Q_{X}Q_{Y}\right)=D_{\alpha }\left(P_{X,Y}∥P_{X}P_{Y}\right)=S\left(X\right)-S\left(X|Y\right) \end{array}$$

where S(X|Y) stands for the Shannon conditional entropy,

(58) $$S\left(X|Y\right)=\sum_{x,y}P_{X,Y}\left(x,y\right)\log P_{X|Y}\left(x|y\right).$$

All of these measures are consistent with the Shannon case in view of the property ($A_{6}$), but their direct usage as measures of Rényi information transfer leads to a breaking of some the properties ($A_{1}$)–($A_{5}$), which justifies the usage of Arimoto’s measures from the previous section as appropriate ones in the context of this research. In the following section, we review the alternative definitions.

### 5.1. Information Transfer Measures by Sibson

Alternative approaches based on Rényi divergence were proposed by Sibson [23] and considered later by several authors in the context of quantum secure communications [39,40,41,42,43,44], who introduced

(59) $$J_{\alpha }^{1}\left(X;Y\right)=\min_{Q_{Y}}D_{\alpha }\left(P_{Y|X}P_{X}∥Q_{Y}P_{X}\right),$$

which can be represented as in [26]

(60) $$J_{\alpha }^{1}\left(X,Y\right)=\frac{\alpha }{\alpha -1}\log\left(\sum_{y}{\left(\sum_{x}P_{X}\left(x\right)P_{Y|X}^{\alpha }\left(y|x\right)\right)}^{\frac{1}{\alpha }}\right)$$

and, in the discrete setting, can be related to the Gallager exponent as in [29]:

(61) $$J_{\alpha }^{1}\left(X,Y\right)=\frac{\alpha }{1-\alpha }E_{0}\left(\frac{1}{\alpha }-1,P_{X}\right),$$

which differs from Arimoto’s definition (61) since in this case the escort distribution does not participate in the error exponent, but an ordinary one does. However, in the case of a perfect channel for which X=Y, the conditional distribution $P_{Y|X}^{\alpha }\left(y|x\right)=1$ for x=y and zero otherwise, so Sibson’s measure (60) reduces to $R_{1/\alpha }\left(X\right)$, thus breaking the axiom ($A_{3}$). This disadvantage can be overcome by the reparametrization α↔1/α so that $J_{1/\alpha }^{1}\left(X,Y\right)$ is used as a measure of Rényi information transfer, and the properties of the resulting measure can be considered in a manner similar to the case of Arimoto.

### 5.2. Information Transfer Measures by Augustin and Csiszar

An alternative definition of Rényi mutual information was also presented by Augustin [25], and later Csiszar [26], who defined

(62) $$J_{\alpha }^{2}\left(X;Y\right)=\min_{Q_{Y}}E\left[D_{\alpha }\left(P_{Y|X}∥Q_{Y}\right)\right],$$

However, in the case of perfect transmission, for which X=Y, the measure reduces to Shannon entropy

(63) $$J_{\alpha }^{2}\left(X;Y\right)=S\left(X\right),$$

which breaks the axiom ($A_{3}$).

### 5.3. Information Transfer Measures by Lapidoth, Pfister, Tomamichel and Hayashi

A similar obstacle to the case of the Augustin–Csiszar measure can be observed in the case of mutual information which was considered by Lapidoth and Pfister [27] and Tomamichel and Hayashi [28], who proposed

(64) $$J_{\alpha }^{3}\left(X;Y\right)=\min_{Q_{X}}\min_{Q_{Y}}D_{\alpha }\left(P_{X,Y}∥Q_{X}Q_{Y}\right).$$

As shown in [27] (Lemma 11), if X=Y, then

(65) $$J_{\alpha }^{3}\left(X;Y\right)=\left\{\begin{array}{cc} \frac{\alpha }{1-\alpha }\lim_{\alpha \to \infty }R_{\alpha }\left(X\right) & \;\mathrm{if}\;\alpha \in \left[0,\frac{1}{2}\right],\\ R_{\frac{\alpha }{2\alpha -1}}\left(X\right) & \;\mathrm{if}\;\alpha >\frac{1}{2} \end{array}\right.$$

so the axiom ($A_{3}$) is broken in this case, as well.

**Remark** **1.** *Despite the difference between the definitions of information transfer, in the discrete setting, the alternative definitions discussed above reach the same maximum over the set of input probability distributions, $P_{X}$,^26^,^29^,^45^].*

### 5.4. Information Transfer Measures by Chapeau-Blondeau, Delahaies, Rousseau, Tridenski, Zamir, Ingber and Harremoes

Chapeau-Blondeau, Delahaies and Rousseau [31], and independently Tridenski, Zamir and Ingber [46] and Harremoes [47], defined the Rényi mutual information using the Rényi divergence (55), so that the mutual information defined using the Rényi divergence

(66) $$J_{\alpha }^{4}\left(X,Y\right)=D_{\alpha }\left(P_{X,Y}∥P_{X}P_{Y}\right)$$

for α>0 and α≠1, while in the case of α=1 it reduces to Shannon mutual information. However, the ordinal definition can correspond only to a Rényi entropy of order 2−α since in the case of X=Y it reduces to $J_{\alpha }^{4}\left(X,Y\right)=R_{2-\alpha }\left(X\right)$ (see also [47]), which can be overcome by the reparametrization α=2−q, similar to the case of Sibson’s measure. This measure has been discussed in the past with various operational characterizations, and could also be considered as a measure of information transfer, although the satisfaction of all of the axioms ($A_{1}$)–($A_{6}$) is not self-evident for general channels.

### 5.5. Information Transfer Measures by Jizba, Kleinert and Shefaat

Finally, we will mention the definition by Jizba, Kleinert and Shefaat [48],

(67) $$J_{\alpha }^{4}\left(X,Y\right)\equiv R_{\alpha }\left(X\right)-{\hat{R}}_{\alpha }\left(X|Y\right),$$

which is defined in the same manner as in Arimoto’s case (42), but with another choice of conditional Rényi entropy

(68) $${\hat{R}}_{\alpha }\left(X|Y\right)=\frac{1}{1-\alpha }\log\sum_{x}P_{X}^{\left(\alpha \right)}\left(x\right)2^{\left(1-\alpha \right)R_{\alpha }\left(X|Y=y\right)},$$

which arises from the Generalized Shannon–Khinchin axiom [GSK4] if the pseudo-additivity in the equation (9) is restricted to an ordinary addition, in which case the GSK axioms uniquely determine Rényi entropy [49]. However, despite its wide applicability in the modeling of causality and financial time series, this mutual information can take negative values which breaks the axiom ($A_{1}$), which is assumed to be mandatory in this paper. For further discussion of the physicalism of negative mutual information in the domain of financial time series analysis, the reader is referred to [48].

## 6. The *α*-*q* Mutual Information and the *α*-*q*-Capacity

In the past several attempts have been done to define an appropriate channel capacity measure which corresponds to instances of the Sharma–Mittal entropy class. All of them follow a similar recipe by which the channel capacity is defined as in (32), as a maximum of appropriately defined mutual information $I_{\alpha ,q}$. However, all of the classes consider only special cases of Sharma–Mittal entropy and all of them fail to satisfy at least one of the properties (${\bar{A}}_{1}$)–(${\bar{A}}_{6}$) which an information transfer has to satisfy, as will be discussed Section 7.

In this section we propose a general measures of the α-*q* mutual information and the α-*q*-capacity by the requirement that the axioms (${\bar{A}}_{1}$)–(${\bar{A}}_{6}$) are satisfied, which could qualify them as appropriate measures of information transfer, without nonphysical properties. The special instances of the α-*q* (maximal) information transfer measures are also discussed and the analytic expressions for a binary symmetric channel are provided.

### 6.1. The α-q Information Transfer Measures and Its Instances

The α-*q*-mutual information (42) is defined using the *q*-subtraction defined in (6), as follows:

(69) $$I_{\alpha ,q}\left(X,Y\right)=H_{\alpha ,q}\left(X\right){⊖}_{q}H_{\alpha ,q}\left(X|Y\right),$$

where we introduced the conditional Sharma–Mittal entropy $H_{\alpha ,q}\left(Y|X\right)$ as in

(70) $$H_{\alpha ,q}\left(X|Y\right)=\eta_{q}\left(R_{\alpha }\left(X|Y\right)\right)=\frac{1}{(1-q)\ln2}\left({\left(\sum_{y}P_{Y}\left(y\right){\left(\sum_{x}P_{X|Y=y}{\left(x\right)}^{\alpha }\right)}^{\frac{1}{\alpha }}\right)}^{\frac{\alpha (1-q)}{\alpha -1}}-1\right),$$

$R_{\alpha }\left(X|Y\right)$ stands for Arimoto’s definition of the conditional Rényi entropy (41). The expression (69) can also be obtained if the mapping $\eta_{q}$ is applied to both sides of the equality (42), by which Arimoto’s mutual information is defined, so we may establish the relationship

(71) $$I_{\alpha ,q}\left(X,Y\right)=\eta_{q}\left(I_{\alpha }\left(X,Y\right)\right)=\eta_{q}\left(\frac{\alpha }{1-\alpha }\log\left(\sum_{y}{\left(\sum_{x}P_{X}^{\left(\alpha \right)}\left(x\right)P_{Y|X}^{\alpha }\left(y|x\right)\right)}^{\frac{1}{\alpha }}\right)\right),$$

which can be represented using the Gallager error exponent (43) as in

(72) $$I_{\alpha ,q}\left(X,Y\right)=\eta_{q}\left(\frac{\alpha }{1-\alpha }E_{0}\left(\frac{1}{\alpha }-1,P_{X}^{\left(\alpha \right)}\right)\right)=\frac{1}{(1-q)\ln2}\left(2^{\frac{\alpha (1-q)}{1-\alpha }E_{0}\left(\frac{1}{\alpha }-1,P_{X}^{\left(\alpha \right)}\right)}-1\right).$$

Arimoto’s α-*q*-capacity is now defined in

(73) $$C_{\alpha ,q}=\max_{P_{X}}I_{\alpha ,q}\left(X,Y\right),$$

and using the fact that $\eta_{q}$ is increasing, it can be related with the corresponding α-capacity as in

(74) $$C_{\alpha ,q}=\max_{P_{X}}I_{\alpha ,q}\left(X,Y\right)=\max_{P_{X}}\eta_{q}\left(I_{\alpha }\left(X,Y\right)\right)=\eta_{q}\left(\max_{P_{X}}I_{\alpha }\left(X,Y\right)\right)=\eta_{q}\left(C_{\alpha }\left(P_{Y|X}\right)\right).$$

Using the expressions (45) and (71), in the case of α=1, the α-*q* mutual information reduces to

(75) $$\begin{array}{cc} I_{1,q} & =\frac{1}{(1-q)\ln2}\left(\underset{x,y}{\Pi }2^{P_{X,Y}\left(x,y\right)\log\frac{P_{X,Y}\left(x,y\right)}{P_{X}\left(x\right)P_{Y}\left(y\right)}}-1\right)\\ \; & =\frac{1}{(1-q)\ln2}\left(\underset{x,y}{\Pi }{\left(\frac{P_{X,Y}\left(x,y\right)}{P_{X}\left(x\right)P_{Y}\left(y\right)}\right)}^{P_{X,Y}\left(x,y\right)}-1\right). \end{array}$$

The α-*q*-capacity is given in

(76) $$C_{1,q}=\max_{P_{X}}\left(\frac{1}{(1-q)\ln2}\left(\underset{x,y}{\Pi }{\left(\frac{P_{X,Y}\left(x,y\right)}{P_{X}\left(x\right)P_{Y}\left(y\right)}\right)}^{P_{X,Y}\left(x,y\right)}-1\right)\right)$$

and these measures can serve as (maximal) information transfer measures corresponding to Gaussian entropy, which was not considered before in the context of information transmission. Naturally, if in addition q→1, the measures reduce to Shannon’s mutual information and Shannon capacity [37].

Additional special cases of the α-*q* (maximal) information transfer include the α-mutual information (42) and the α-capacity (40), which are obtained for q=1; the measures which correspond to Tsallis entropy can be obtained for q=α and the ones which correspond to Landsberg–Vedral entropy for q=2−α. These special instances are listed in Table 1.

As discussed in Section 7, previously considered information measures cover only particular special cases and break at least one of the axioms (${\bar{A}}_{1}$)–(${\bar{A}}_{5}$), which leads to unexpected and counterintuitive conclusions about the channels, such as negative information transfer and achieving super-capacitance or sub-capacitance [4], which could be treated as a nonphysical behavior. On the other hand, apart from the generality, the α-*q* information transfer measures proposed in this paper overcame the disadvantages which could qualify them as appropriate measures, as stated in the following theorem.

**Theorem** **2.** *The α-q information transfer measures $I_{\alpha ,q}$ and $C_{\alpha ,q}$ satisfy the set of the axioms (${\bar{A}}_{1}$)–(${\bar{A}}_{6}$).*

**Proof.**  The proof is the straightforward application of the mapping $\eta_{q}$ to the equations in the α-mutual information properties ($A_{1}$)–($A_{5}$), while the (${\bar{A}}_{6}$) follows from the above discussion. □

**Remark** **2.** *Note that the symmetry $I_{\alpha ,q}\left(X,Y\right)=I_{\alpha ,q}\left(Y,X\right)$ does not hold in general in the case of the α-q mutual information nor in the case of the α mutual information [50,51] and if the mutual information is defined so that the symmetry is preserved, some of the axioms (${\bar{A}}_{1}$)–(${\bar{A}}_{6}$) might be broken. In addition, the alternative definition of the mutual information, $I_{\alpha ,q}\left(Y,X\right)=H_{\alpha ,q}\left(Y\right)-H_{\alpha ,q}\left(Y|X\right)$, which uses an ordinary substraction operator instead of ${⊖}_{q}$ operation, can also be introduced, but in this case the property (${\bar{A}}_{5}$) might not hold in general, as discussed in Section 7.*

### 6.2. The α-q-Capacity of Binary Symmetric Channels

As shown by Cai and Verdú [45], the α-mutual information of Arimoto’s type $I_{\alpha }$ is maximized for the uniform distribution $P_{X}=\left(1/2,1/2\right)$, and Arimoto’s α-capacity has the value

(77) $$C_{\alpha }\left(BSC\right)=1-r_{\alpha }\left(p\right),$$

where the binary entropy function $r_{\alpha }$ is defined as

(78) $$r_{\alpha }\left(p\right)=R_{\alpha }\left(p,1-p\right)=\frac{1}{1-\alpha }\log\left(p^{\alpha }+{\left(1-p\right)}^{\alpha }\right),$$

for α>0, α≠1, while in the limit of α→1, the expression (78) reduces to the well-known result for the Shannon capacity (see Fano [52])

(79) $$C_{1}\left(BSC\right)=\lim_{\alpha \to 1}C_{\alpha }\left(BSC\right)=1+p\log p+\left(1-p\right)\log\left(1-p\right).$$

The analytic expressions for the α-*q*-capacities of binary symmetric channel’s can be obtained from the expressions (74) and (77), so that

(80) $$C_{\alpha ,q}\left(BSC\right)=q\left(C_{\alpha }\left(BSC\right)\right)=\frac{1}{(1-q)\ln2}\left(2^{1-q}{\left(p^{\alpha }+{\left(1-p\right)}^{\alpha }\right)}^{-\frac{1-q}{1-\alpha }}-1\right);$$

in the case of q=1, it reduces to the case of Rényi entropy while, in the case of α=1, to the case of Gaussian entropy (77)

(81) $$C_{1,q}\left(BSC\right)=\frac{1}{(1-q)\ln2}\left(2p^{p}{\left(1-p\right)}^{1-p}-1\right).$$

**Figure 3:**
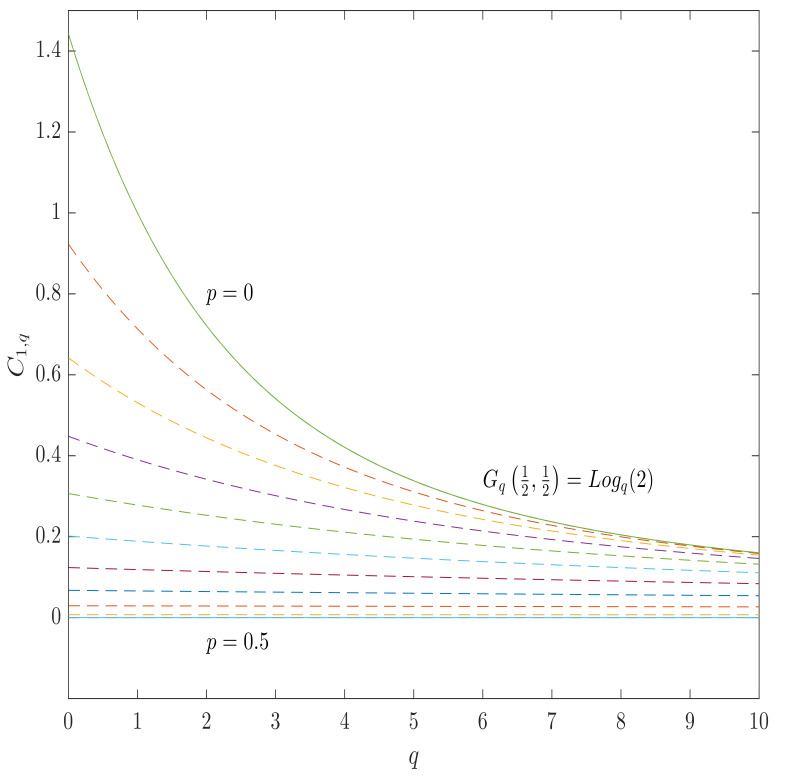
The α-*q*-capacity of BSC for the Gaussian entropy (the case of α=1) as a function of *q* for various values of the channel parameter *p* from 0.5 (totally destructive channel) to 0 (perfect transmission). All of the curves lies between 0 and ${\mathrm{Log}}_{q}2$, which is the maximum value of the Gaussian entropy.

**Figure 4:**
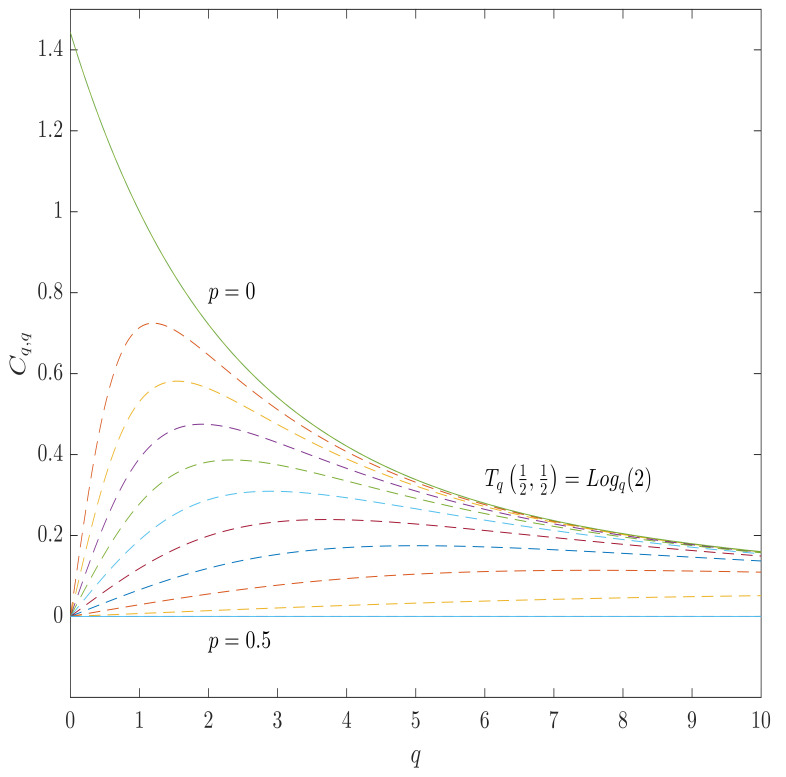
The α-*q*-capacity of BSC for the Tsallis entropy (the case of α=q) as a function of *q* for various values of the channel parameter *p* from 0.5 (totally destructive channel) to 0 (perfect transmission). All of the curves lies between 0 and ${\mathrm{Log}}_{q}2$, which is the maximum value of the Tsallis entropy.

The analytic expressions for BSC α-*q* capacities for other instances can straightforwardly be obtained by specifying the values of the parameters, whose instances are listed in Table 1, while the plots of the BSC α-*q*-capacities, which correspond to the Gaussian and the Tsallis entropies, are shown in Figure 3 and Figure 4.

The α-*q*-capacity (80) can equivalently be expressed in

(82) $$C_{\alpha ,q}\left(BSC\right)={\mathrm{Log}}_{q}\;2{⊖}_{q}h_{\alpha ,q}\left(p\right),$$

where the Sharma–Mittal binary entropy function is defined in

(83) $$h_{\alpha ,q}\left(p\right)=H_{\alpha ,q}\left(p,1-p\right)=\frac{1}{1-q}\left({\left(p^{\alpha }+{\left(1-p\right)}^{\alpha }\right)}^{\frac{1-q}{1-\alpha }}-1\right),$$

which reduces to the Rényi binary entropy function, in the case of q=1,

(84) $$h_{\alpha ,1}\left(p\right)=\lim_{q\to 1}h_{\alpha ,q}\left(p\right)=R_{\alpha }\left(p,1-p\right)=\frac{1}{1-\alpha }\log\left(p^{\alpha }+{\left(1-p\right)}^{\alpha })\right),$$

to the Tsallis binary entropy function, in the case of α=1,

(85) $$h_{q,q}\left(p\right)=h_{q,q}\left(p\right)=T_{q}\left(p,1-p\right)=\frac{1}{1-q}\left(p^{q}+{\left(1-p\right)}^{q}-1\right),$$

to the Gaussian binary entropy function, in the case of α=1,

(86) $$h_{1,q}\left(p\right)=\lim_{\alpha \to 1}h_{\alpha ,q}\left(p\right)=G_{q}\left(p,1-p\right)=\frac{1}{(1-q)\ln2}\left(p^{-(1-q)p}{\left(1-p\right)}^{-(1-q)\left(1-p\right)}-1\right),$$

and to the Shannon binary entropy function, in the case of α=q=1,

(87) $$h_{1,1}\left(p\right)=\lim_{q,\alpha \to 1}h_{\alpha ,q}\left(p\right)=S\left(p,1-p\right)=-p\log p-\left(1-p\right)\log\left(1-p\right).$$

The expression (82) can be interpreted similarly as in the Shannon case. Thus, a BSC channel with input *X* and output *Y* can be modeled with an input–output relation Y=X⊕Z where ⊕ stands for modulo 2 sum and *Z* is channel noise taking values from {1,0}, distributed in accordance with (p,1−p). If we measure the information which is lost per bit during transmission with the Sharma–Mittal entropy $H_{\alpha ,q}\left(Z\right)=h_{\alpha }\left(p\right)$, then $C_{\alpha ,q}$ stands for useful information left over for every bit of information received.

## 7. An Overview of the Previous Approaches to Sharma–Mittal Information Transfer Measures

In this section, we review the previous attempts at a definition of Sharma–Mittal information transfer measures, which are defined from the basic requirement of consistency with the Shannon measure as given by the axiom (${\bar{A}}_{6}$). However, as we show in the following paragraphs, all of them break at least one of the axioms (${\bar{A}}_{1}$)–(${\bar{A}}_{5}$), which are satisfied in the case of the α-*q* (maximal) information transfer measures (69) and (73), in accordance with the discussion in Section 6.

### 7.1. Daróczy’s Capacity

The first considerations of generalized channel capacities and generalized mutual information for the *q*-entropy go back to Daróczy [30], who introduced conditional Tsallis entropy

(88) $${\bar{T}}_{q}\left(Y|X\right)=\sum_{x}P_{X}^{q}\left(x\right)T_{q}\left(Y|X=x\right),$$

where the row entropies are defined as in

(89) $$T_{q}\left(Y|X=x\right)=\frac{1}{\left(1-q)\log(2\right)}\left(\sum_{x}P_{Y|X}{\left(y|x\right)}^{q}-1\right)$$

and the mutual information is defined as in

(90) $$J_{\alpha ,q}^{5}\left(X,Y\right)=T_{q}\left(Y\right)-{\bar{T}}_{q}\left(Y|X\right).$$

However, in the case of a totally destructive channel, X⊥⊥Y, $P_{Y|X}\left(y|x\right)=P_{Y}\left(y\right)$, $T_{q}\left(Y|X=x\right)=T_{q}\left(Y\right)$ and

(91) $$T_{q}\left(Y|X\right)=T_{q}\left(Y\right)\sum_{x}P_{X}{\left(x\right)}^{q}$$

so that

(92) $$J_{\alpha ,q}^{5}\left(X,Y\right)=T_{q}\left(Y\right)\left(1-\sum_{x}P_{X}{\left(x\right)}^{q}\right)=\left(1-\sum_{x}P_{X}{\left(x\right)}^{q}\right){\mathrm{Log}}_{q}m.$$

**Figure 5:**
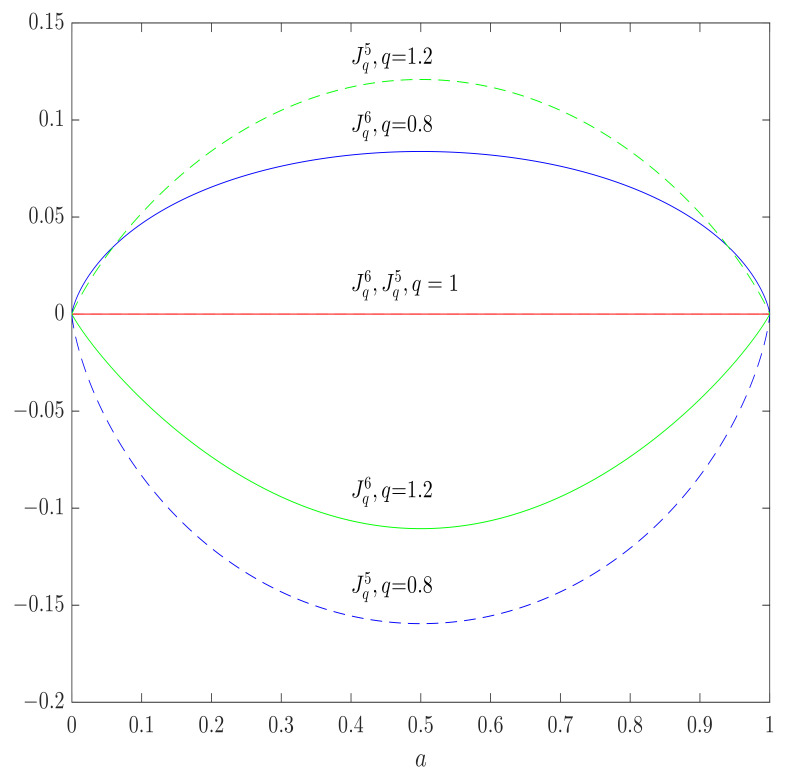
Daróczy’s (solid lines) and Yamano’s (dashed lines) mutual information in the case of a totally destructive BSC as functions of the input distribution parameter *a*, $P_{X}={\left[a,1-a\right]}^{T}$ for different values of *q*, obtaining negative values for q<1 and q>1, respectively, breaking the axioms (${\bar{A}}_{1}$) and (${\bar{A}}_{2}$). The α-*q*-mutual information is zero; for all *q*, and satisfies (${\bar{A}}_{1}$) and (${\bar{A}}_{2}$).

**Figure 6:**
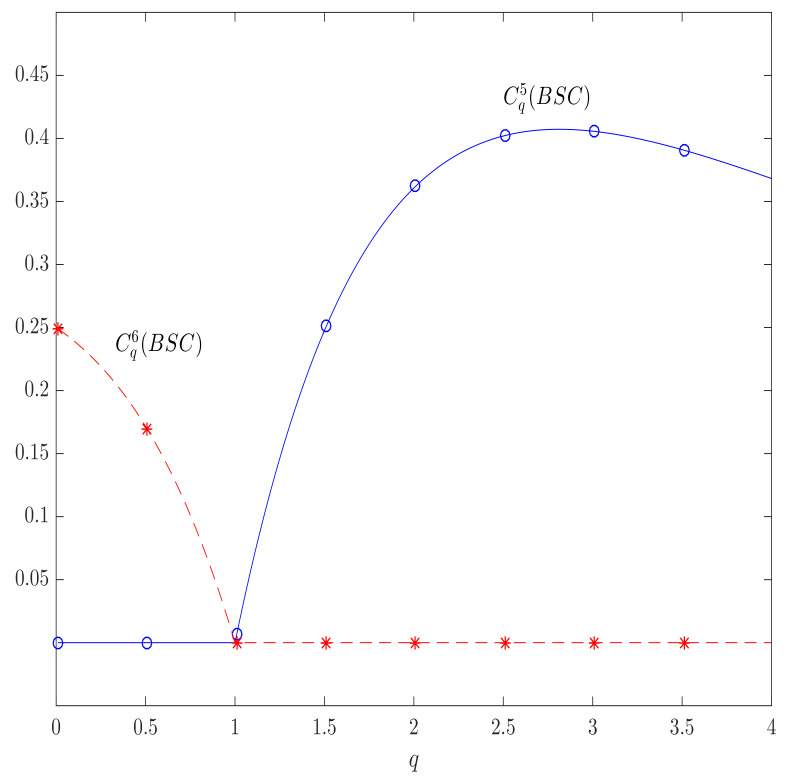
Daróczy’s (solid lines) and Yamano’s (dashed lines) capacities in the case of totally destructive BSC as functions of the parameter *q*. In the regions of q<1 and q>1, respectively, the corresponding negative mutual information is maximized for $P_{X}={\left[1,0\right]}^{T}$ (zero capacity) having the positive values outside the regions and breaking the axiom (${\bar{A}}_{2}$). The α-*q*-capacity is zero; for all *q*, and satisfies (${\bar{A}}_{2}$).

This expression is zero for an input probability distribution $P_{X}=\left(1,0,\ldots ,0\right)$ and its permutations, but, in general, it is negative for q<1, positive for q>1 and 0 only for q=1, so the axiom (${\bar{A}}_{2}$) is broken (see Figure 5). As a result, the channel capacity, which is defined in accordance to (32), is zero for q≤1 and positive for q>1, as illustrated in Figure 6 by the example of BSC for which the Daroczy’s channel capacity can be computed as in [30,53]

(93) $$C_{q}^{5}\left(BSC\right)=\frac{1-2^{1-q}}{q-1}-\frac{2^{-q}}{q-1}\left[1-{\left(1-p\right)}^{q}-p^{q}\right].$$

In the same figure, we plotted the graph for the α-*q* channel capacities proposed in this paper, and all of them remain zero in the case of a totally destructive BSC, as expected.

### 7.2. Yamano Capacities

Similar problems to the ones mentioned above arise in the case of mutual information and corresponding capacity measures considered by Yamano [33], who addressed the information transmission characterized by Landsberg–Vedral entropy $L_{q}$, given in (17).

Thus, the first proposal is based on the mutual information of the form

(94) $$J_{q}^{6}\left(X,Y\right)=L_{q}\left(X\right)+L_{q}\left(Y\right)-L_{q}\left(X,Y\right),$$

where the joint entropy is defined in

(95) $$L_{q}\left(X,Y\right)=\frac{1}{q-1}\left(\frac{1}{\sum_{x,y}P_{X,Y}{\left(x,y\right)}^{q}}-1\right).$$

However, in the case of a fully destructive channel, $P_{Y}\left(y\right)=1/m$ and $P_{X,Y}\left(x,y\right)=P_{X}\left(x\right)/m$, so that

(96) $$J_{q}^{6}\left(X,Y\right)=\frac{1}{q-1}\left(\frac{1}{\sum_{x}P_{X}{\left(x\right)}^{q}}-1\right)+\frac{1}{q-1}\left(m^{q-1}-1\right)-\frac{1}{q-1}\left(m^{q-1}\frac{1}{\sum_{x}P_{X}{\left(x\right)}^{q}}-1\right),$$

which can be simplified to

(97) $$J_{q}^{6}\left(X,Y\right)=\frac{1-m^{q-1}}{q-1}\left(\frac{1}{\sum_{x}P_{X}{\left(x\right)}^{q}}-1\right).$$

Similarly to the case of Daroczy’s capacity, this expression is zero for an input probability distribution $P_{X}=\left(1,0,\ldots ,0\right)$ and its permutations but, in general, it is negative for q>1, positive for q<1 and 0 only for q=1, so the axiom (${\bar{A}}_{2}$) is broken (see Figure 5). In Figure 6 we illustrated the Yamano channel capacity as a function of the parameter *q*, in the case of two input channels with $P_{X}=\left[a,1-a\right]$, the channel capacity is zero for q>1 (which is obtained for $P_{X}=\left[1,0\right]$), and

(98) $$C_{q}^{6}\left(BSC\right)=\frac{1}{q-1}\left(2^{q}-1-2^{2q-2}\right),$$

for q>1 (which is obtained for $P_{X}=\left[1/2,1/2\right]$). In the same Figure, we plotted the graph for the α-*q* channel capacities proposed in this paper, and, as before, all of them remain zero in the case of a totally destructive BSC, as expected.

Further attempts were made in [33], where the mutual information is defined in an analogous manner to (66) and (66), with the generalized divergence measure introduced in [54]. Thus, the alternative measure for mutual information is defined in

(99) $$\begin{array}{c} J_{q}^{7}\left(X,Y\right)=\frac{1}{(1-q)\ln2}\frac{1}{\sum_{x,y}P_{X,Y}^{q}\left(x,y\right)}\left[1-\sum_{x,y}P_{X,Y}\left(x,y\right){\left(\frac{P_{X}\left(x\right)P_{Y}\left(y\right)}{P_{X,Y}\left(x,y\right)}\right)}^{1-q}\right]. \end{array}$$

However, in the case of the simplest perfect communication channel for which X=Y, the mutual information reduces to

(100) $$J_{q}^{7}\left(X,Y\right)=\frac{1}{(1-q)\ln2}\frac{1-\sum_{x}P_{X}{\left(x\right)}^{2-q}}{\sum_{x}P_{X}{\left(x\right)}^{q}}\neq L_{q}\left(X\right),$$

which breaks the axiom (${\bar{A}}_{3}$).

### 7.3. Landsber–Vedral Capacities

To avoid these problems, Landsberg and Vedral [4] proposed the mutual information measure and related channel capacities for the Sharma–Mittal entropy class $H_{\alpha ,q}$, particularly considering the choice of q=α, which corresponds to Tsallis entropy, q=2−α, and the case of q=1, which corresponds to the Rényi entropy

(101) $$J_{\alpha ,q}^{8}\left(X,Y\right)=H_{\alpha ,q}\left(Y\right)-\tilde{H_{\alpha ,q}}\left(Y|X\right),$$

where the conditional entropy ${\tilde{H_{\alpha ,q}}}^{LV}\left(Y|X\right)$ is defined as in

(102) $$\tilde{H_{\alpha ,q}}\left(Y|X\right)=\sum_{x}P_{X}\left(x\right)H_{\alpha ,q}\left(Y|X=x\right)$$

and

(103) $$H_{\alpha ,q}\left(Y|X=x\right)=\frac{1}{1-q}\left({\left(\sum_{y}P_{Y|X}{\left(y|x\right)}^{\alpha }\right)}^{\frac{1-q}{1-\alpha }}-1\right).$$

Although this definition bears some similarities to the α-*q* mutual information proposed in formula (69), several key differences can be observed. First of all, it characterizes the information transfer as the output uncertainty reduction after the input symbols are known, instead of input uncertainty reduction, after the output symbols are known (42). In addition, it uses the ordinary—operation instead of the ${⊖}_{q}$ one. In addition, note that the definition of conditional entropy (102) generally differs from the definition proposed in (70).

**Figure 7:**
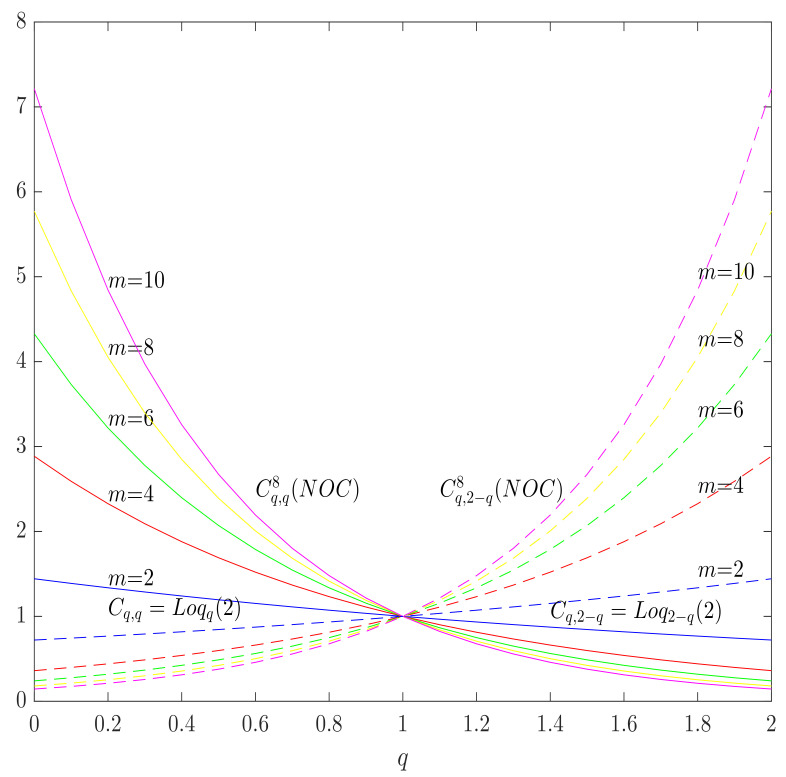
Landsberg–Vedral capacities for the Tsallis (solid lines) and the Landsberg–Vedral (dashed lines) entropies in the case of a (perfect) noisy channel with non-overlapping outputs with *m* outputs as functions of *q*, for different values of *m*. The axiom (${\bar{A}}_{4}$) is broken for all m>2 and satisfied in the case of corresponding α-*q*-capacities, $C_{q,q}$ and $C_{q,2-q}$.

The definition (101) resolves the issue of the axiom (${\bar{A}}_{2}$) which appears in the case of the Daroczy capacity, since in the case of a totally destructive channel (X⊥⊥Y), $P_{Y|X}\left(y|x\right)=P_{Y}\left(y\right)$ and $L_{q}\left(Y|X=x\right)=L_{q}\left(Y\right)$ and $L_{q}\left(Y|X\right)=L_{q}\left(Y\right)$, so that $I_{\alpha ,q}^{lv}\left(X,Y\right)=0$. However, the problems remain with the axiom (${\bar{A}}_{5}$), which can be observed in the case of a noisy channel with non-overlapping outputs if the number of channel inputs is lower than the number of channel outputs n<m. Indeed, in the case of a noisy channel with non-overlapping outputs given by the transition matrix (27), both of the row entropies $L_{q}\left(Y|X=x\right)$ have the same value, which is independent of *x*

(104) $$H_{\alpha ,q}\left(Y|X=x\right)=\frac{k^{1-q}-1}{(q-1)\ln2}={\mathrm{Log}}_{q}k;\;\mathrm{for}\;x=x_{1},x_{2},$$

and the maximal value of Landsberg–Vedral mutual information (101) is obtained only by maximizing $H_{\alpha ,q}\left(Y\right)$ over $P_{X}$, which is achieved if *X* is uniformly distributed, since in this case *Y* is uniformly distributed, as well as ($a=\frac{1}{2}$ in (28)), so the maximal value of the output entropy is $H_{\alpha ,q}\left(Y\right)={\mathrm{Log}}_{q}\left(2k\right)$ and the mutual information is maximized for

(105) $$C_{\alpha ,q}^{8}\left(NOC\right)={\mathrm{Log}}_{q}\left(2k\right)-{\mathrm{Log}}_{q}\left(k\right),$$

which is greater than ${\mathrm{Log}}_{q}\left(2\right)$ for k≥2, i.e., for m≥4 outputs, so the axiom (${\bar{A}}_{5}$) is broken, which is illustrated in Figure 7.

### 7.4. Chapeau-Blondeau–Delahaies–Rousseau Capacities

Following a similar approach to the one in Section 5.4, Chapeau-Blondeau, Delahaies and Rousseau considered the definition of mutual information which corresponds to the Tsallis entropy using Tsallis divergence,

(106) $$D_{q,q}\left(P||Q\right)=\frac{1}{q-1}\left(\sum_{x}P{\left(x\right)}^{q}Q{\left(x\right)}^{1-q}-1\right),$$

can be written in

(107) $$\begin{array}{cc} J_{q}^{9}\left(X,Y\right) & =D_{q,q}\left(P_{X,Y}∥P_{X}P_{Y}\right)=\eta_{q}\left(D_{q}\left(P_{X,Y}∥P_{X}P_{Y}\right)\right)\\ \; & =\frac{1}{1-q}\left(1-\sum_{x,y}P_{X,Y}{\left(x,y\right)}^{q}P_{X}{\left(x\right)}^{1-q}P_{Y}{\left(y\right)}^{1-q}\right). \end{array}$$

However, this definition is not directly applicable as a measure of information transfer to the Tsallis entropy with index *q*, since in the case of X=Y it reduces to $J_{q}^{9}\left(X,Y\right)=T_{2-q}\left(X\right)$, and requires the reparametrization q↔2−q, similar to Section 5.4, while the satisfaction of the axioms (${\bar{A}}_{4}$) and (${\bar{A}}_{5}$) is not self evident.

## 8. Conclusions and Future Work

A general treatment of the Sharma–Mittal entropy transfer was provided together with the analyses of existing information transfer measures for the non-additive Sharma–Mittal information transfer. It was shown that the existing definitions fail to satisfy at least one of the axioms common to the Shannon case, by which the information transfer has to be non-negative, less than the input and output uncertainty, equal to the input uncertainty in the case of perfect transmission and equal to zero in the case of a totally destructive channel. Thus, breaking some of these axioms implies unexpected and counterintuitive conclusions about the channels, such as achieving super-capacitance or sub-capacitance [4], which could be treated as nonphysical behavior. In this paper, alternative measures of the α-*q* mutual information and the α-*q* channel capacity were proposed so that all of the axioms which are broken in the case of the Sharma–Mittal information transfer measures considered before are satisfied, which could qualify them as physically consistent measures of information transfer.

Taking into account the previous research of non-extensive statistical mechanics [3], where the linear growth of the physical quantities has been recognized as a critical property in non-extensive [55] and non-exponentially growing systems [56], and taking into account the previous research from the field of information theory, where the Sharma–Mittal entropy has been considered an appropriate scaling measure which provides extensive information rates [21], the α-*q* mutual information and the α-*q* channel capacity seem to be promising measures for the characterization of information transmission in the systems where the Shannon entropy rate diverges or disappears in an infinite time limit. In addition, as was shown in this paper, the proposed information transfer measures are compatible with the maximum likelihood detection, which indicates their potential for operational characterization of coding theory and hypothesis testing problems [26].

## Figures and Tables

**Table 1:** Instances of the α-*q*-mutual information for different values of the parameters and corresponding expressions for the BSC α-*q*-capacities.

$H_{\alpha ,q}$	$I_{\alpha ,q}$	$C_{\alpha ,q}$
*S* α=q=1	$\sum_{x,y}P_{X,Y}\left(x,y\right)\log\frac{P_{X,Y}\left(x,y\right)}{P_{X}\left(x\right)P_{Y}\left(y\right)}$	1+plogp+(1−p)log(1−p)
$R_{\alpha }$ q=1	$\frac{\alpha }{1-\alpha }E_{0}\left(\frac{1}{\alpha }-1,P_{X}^{\left(\alpha \right)}\right)$	$1-\frac{\log(p^{\alpha }+{\left(1-p\right)}^{\alpha })}{1-\alpha }$
$T_{q}$ q=α	$\frac{1}{(1-q)\ln2}\left(2^{qE_{0}\left(\frac{1}{q}-1,P_{X}^{\left(q\right)}\right)}-1\right)$	$\frac{1}{(1-q)\ln2}\left(2^{1-q}{\left(p^{q}+{\left(1-p\right)}^{q}\right)}^{-1}-1\right)$
$L_{\alpha }$ q=2−α	$\frac{1}{(\alpha -1)\ln2}\left(2^{-\alpha E_{0}\left(\frac{1}{\alpha }-1,P_{X}^{\left(\alpha \right)}\right)}-1\right)$	$\frac{1}{(1-\alpha )\ln2}\left(2^{\alpha -1}\left(p^{\alpha }+{\left(1-p\right)}^{\alpha }\right)-1\right)$
$G_{q}$ α=1	$\frac{1}{(1-q)\ln2}\left(\Pi_{x,y}{\left(\frac{P_{X,Y}\left(x,y\right)}{P_{X}\left(x\right)P_{Y}\left(y\right)}\right)}^{P_{X,Y}\left(x,y\right)}-1\right)$	$\frac{1}{(1-q)\ln2}\left(2^{1-q}p^{(1-q)p}{\left(1-p\right)}^{(1-q)\left(1-p\right)}-1\right)$
$E_{0}\left(\rho ,P_{X}\right)=-\log\left(\sum_{y}{\left(\sum_{x}P_{X}\left(x\right)P_{Y|X}^{\frac{1}{1+\rho }}\left(y|x\right)\right)}^{1+\rho }\right)$		

## Data Availability

Not applicable.

## References

[1] Ilić V.M., Stanković M.S. (2014). A unified characterization of generalized information and certainty measures. Phys. A Stat. Mech. Appl..

[2] Renyi A. (1970). Probability Theory.

[3] Tsallis C. (1988). Possible generalization of Boltzmann-Gibbs statistics. J. Stat. Phys..

[4] Landsberg P.T., Vedral V. (1998). Distributions and channel capacities in generalized statistical mechanics. Phys. Lett. A.

[5] Frank T., Daffertshofer A. (2000). Exact time-dependent solutions of the Renyi Fokker-Planck equation and the Fokker-Planck equations related to the entropies proposed by Sharma and Mittal. Phys. A Stat. Mech. Appl..

[6] Sharma B., Mittal D. (1975). New non-additive measures of entropy for discrete probability distributions. J. Math. Sci..

[7] Tsallis C. (1994). What are the numbers that experiments provide. Quim. Nova.

[8] Nivanen L., Le Méhauté A., Wang Q.A. (2003). Generalized algebra within a nonextensive statistics. Rep. Math. Phys..

[9] Ilić V.M., Stanković M.S. (2014). Generalized Shannon-Khinchin axioms and uniqueness theorem for pseudo-additive entropies. Phys. A Stat. Mech. Appl..

[10] Jizba P., Korbel J. (2020). When Shannon and Khinchin meet Shore and Johnson: Equivalence of information theory and statistical inference axiomatics. Phys. Rev. E.

[11] Esteban M.D., Morales D. (1995). A summary on entropy statistics. Kybernetika.

[12] Lenzi E., Scarfone A. (2012). Extensive-like and intensive-like thermodynamical variables in generalized thermostatistics. Phys. A Stat. Mech. Appl..

[13] Frank T., Plastino A. (2002). Generalized thermostatistics based on the Sharma-Mittal entropy and escort mean values. Eur. Phys. J. B Condens. Matter Complex Syst..

[14] Aktürk O.Ü., Aktürk E., Tomak M. (2008). Can Sobolev inequality be written for Sharma-Mittal entropy?. Int. J. Theor. Phys..

[15] Mazumdar S., Dutta S., Guha P. (2019). Sharma–Mittal quantum discord. Quantum Inf. Process..

[16] Elhoseiny M., Elgammal A. (2015). Generalized Twin Gaussian processes using Sharma–Mittal divergence. Mach. Learn..

[17] Koltcov S., Ignatenko V., Koltsova O. (2019). Estimating Topic Modeling Performance with Sharma–Mittal Entropy. Entropy.

[18] Jawad A., Bamba K., Younas M., Qummer S., Rani S. (2018). Tsallis, Rényi and Sharma-Mittal holographic dark energy models in loop quantum cosmology. Symmetry.

[19] Ghaffari S., Ziaie A., Moradpour H., Asghariyan F., Feleppa F., Tavayef M. (2019). Black hole thermodynamics in Sharma–Mittal generalized entropy formalism. Gen. Relativ. Gravit..

[20] Américo A., Khouzani M., Malacaria P. (2020). Conditional Entropy and Data Processing: An Axiomatic Approach Based on Core-Concavity. IEEE Trans. Inf. Theory.

[21] Girardin V., Lhote L. (2015). Rescaling entropy and divergence rates. IEEE Trans. Inf. Theory.

[22] Ciuperca G., Girardin V., Lhote L. (2011). Computation and estimation of generalized entropy rates for denumerable Markov chains. IEEE Trans. Inf. Theory.

[23] Sibson R. (1969). Information radius. Z. Wahrscheinlichkeitstheorie Verwandte Geb..

[24] Arimoto S., Csiszár I., Elias P. (1977). Information Mesures and Capacity of Order *α* for Discrete Memoryless Channels. Topics in Information Theory.

[25] Augustin U. (1978). Noisy Channels. Ph.D. Thesis.

[26] Csiszár I. (1995). Generalized cutoff rates and Rényi’s information measures. IEEE Trans. Inf. Theory.

[27] Lapidoth A., Pfister C. (2019). Two measures of dependence. Entropy.

[28] Tomamichel M., Hayashi M. (2017). Operational interpretation of Rényi information measures via composite hypothesis testing against product and Markov distributions. IEEE Trans. Inf. Theory.

[29] Verdú S. *α*-mutual information. Proceedings of the 2015 Information Theory and Applications Workshop (ITA).

[30] Daróczy Z. (1970). Generalized information functions. Inf. Control.

[31] Chapeau-Blondeau F., Rousseau D., Delahaies A. (2010). Renyi entropy measure of noise-aided information transmission in a binary channel. Phys. Rev. E.

[32] Chapeau-Blondeau F., Delahaies A., Rousseau D. (2011). Tsallis entropy measure of noise-aided information transmission in a binary channel. Phys. Lett. A.

[33] Yamano T. (2001). A possible extension of Shannon’s information theory. Entropy.

[34] Shannon C.E. (1948). A mathematical theory of communication. Bell Syst. Tech. J..

[35] Arimoto S. (1976). Computation of random coding exponent functions. Inf. Theory IEEE Trans..

[36] Gallager R. (1965). A simple derivation of the coding theorem and some applications. IEEE Trans. Inf. Theory.

[37] Cover T.M., Thomas J.A. (2006). Elements of Information Theory (Wiley Series in Telecommunications and Signal Processing).

[38] Fehr S., Berens S. (2014). On the conditional Rényi entropy. Inf. Theory IEEE Trans..

[39] Wilde M.M., Winter A., Yang D. (2014). Strong converse for the classical capacity of entanglement-breaking and Hadamard channels via a sandwiched Rényi relative entropy. Commun. Math. Phys..

[40] Gupta M.K., Wilde M.M. (2015). Multiplicativity of completely bounded p-norms implies a strong converse for entanglement-assisted capacity. Commun. Math. Phys..

[41] Beigi S. (2013). Sandwiched Rényi divergence satisfies data processing inequality. J. Math. Phys..

[42] Hayashi M., Tomamichel M. (2016). Correlation detection and an operational interpretation of the Rényi mutual information. J. Math. Phys..

[43] Hayashi M., Tajima H. (2017). Measurement-based formulation of quantum heat engines. Phys. Rev. A.

[44] Hayashi M. (2015). Quantum Wiretap Channel With Non-Uniform Random Number and Its Exponent and Equivocation Rate of Leaked Information. IEEE Trans. Inf. Theory.

[45] Cai C., Verdú S. (2019). Conditional Rényi Divergence Saddlepoint and the Maximization of *α*-Mutual Information. Entropy.

[46] Tridenski S., Zamir R., Ingber A. (2015). The Ziv–Zakai–Rényi bound for joint source-channel coding. IEEE Trans. Inf. Theory.

[47] Harremoës P. (2006). Interpretations of Rényi entropies and divergences. Phys. A Stat. Mech. Its Appl..

[48] Jizba P., Kleinert H., Shefaat M. (2012). Rényi’s information transfer between financial time series. Phys. A Stat. Mech. Appl..

[49] Jizba P., Arimitsu T. (2004). The world according to Rényi: Thermodynamics of multifractal systems. Ann. Phys..

[50] Iwamoto M., Shikata J. Information theoretic security for encryption based on conditional Rényi entropies. Proceedings of the International Conference on Information Theoretic Security.

[51] Ilić V., Djordjević I., Stanković M. (2018). On a general definition of conditional Rényi entropies. Proceedings.

[52] Fano R.M. (1961). Transmission of Information.

[53] Ilic V.M., Djordjevic I.B., Küeppers F. (2015). On the Daróczy-Tsallis capacities of discrete channels. Entropy.

[54] Yamano T. (2001). Information theory based on nonadditive information content. Phys. Rev. E.

[55] Tsallis C., Gell-Mann M., Sato Y. (2005). Asymptotically scale-invariant occupancy of phase space makes the entropy Sq extensive. Proc. Natl. Acad. Sci. USA.

[56] Korbel J., Hanel R., Thurner S. (2018). Classification of complex systems by their sample-space scaling exponents. New J. Phys..

